# Single-Molecule Enzyme Activity Analysis for Illuminating Pathological Proteoforms

**DOI:** 10.1021/acscentsci.5c00100

**Published:** 2025-06-17

**Authors:** Toru Komatsu, Tadahaya Mizuno

**Affiliations:** Graduate School of Pharmaceutical Sciences, 13143 The University of Tokyo, Tokyo 113-0033, Japan

## Abstract

Functional protein analysis offers unique insights into phenotype-related changes that cannot be fully captured through genetic or transcriptomic data alone. This renewed focus has sparked growing interest in activity-based diagnostics. Conventional methodologies treat 10^6^–10^9^ protein molecules in bulk. Meanwhile, new approaches are emerging to probe the functions of individual protein molecules. In this Outlook, we discuss recent challenges in applying single-molecule enzyme activity assays to understand disease-related alterations in enzyme activity landscapes at the proteoform level, with potential applications in disease diagnosis.

## Graphical abstract

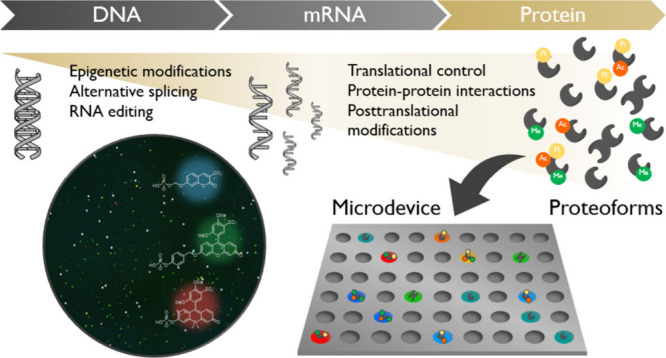

## Introduction

Functional alterations in proteins are strongly associated with disease onset, and understanding these alterations leads to improved diagnosis and treatment.^1,2^ In biological systems, protein function is controlled by various factors at the translational, transcriptional, and post-translational levels, making protein behavior fundamentally different from that of genes and mRNAs.^3^ “Proteoform” refers to different molecular forms of a protein produced via genetic variations, splicing variants, and post-translational modifications (PTMs).^4,5^ Although single gene products can have multiple proteoforms with different functions, conventional assays of protein functions, such as colorimetric/fluorometric assays, mass spectrometry, and antibody-based detection, require bulk analysis of 10^6^–10^9^ protein molecules (attomol to femtomol quantities) per gene product.^6^ Therefore, functional information about important proteoforms can be overlooked in ensemble-averaged data (Figure 1A).

**Figure 1:**
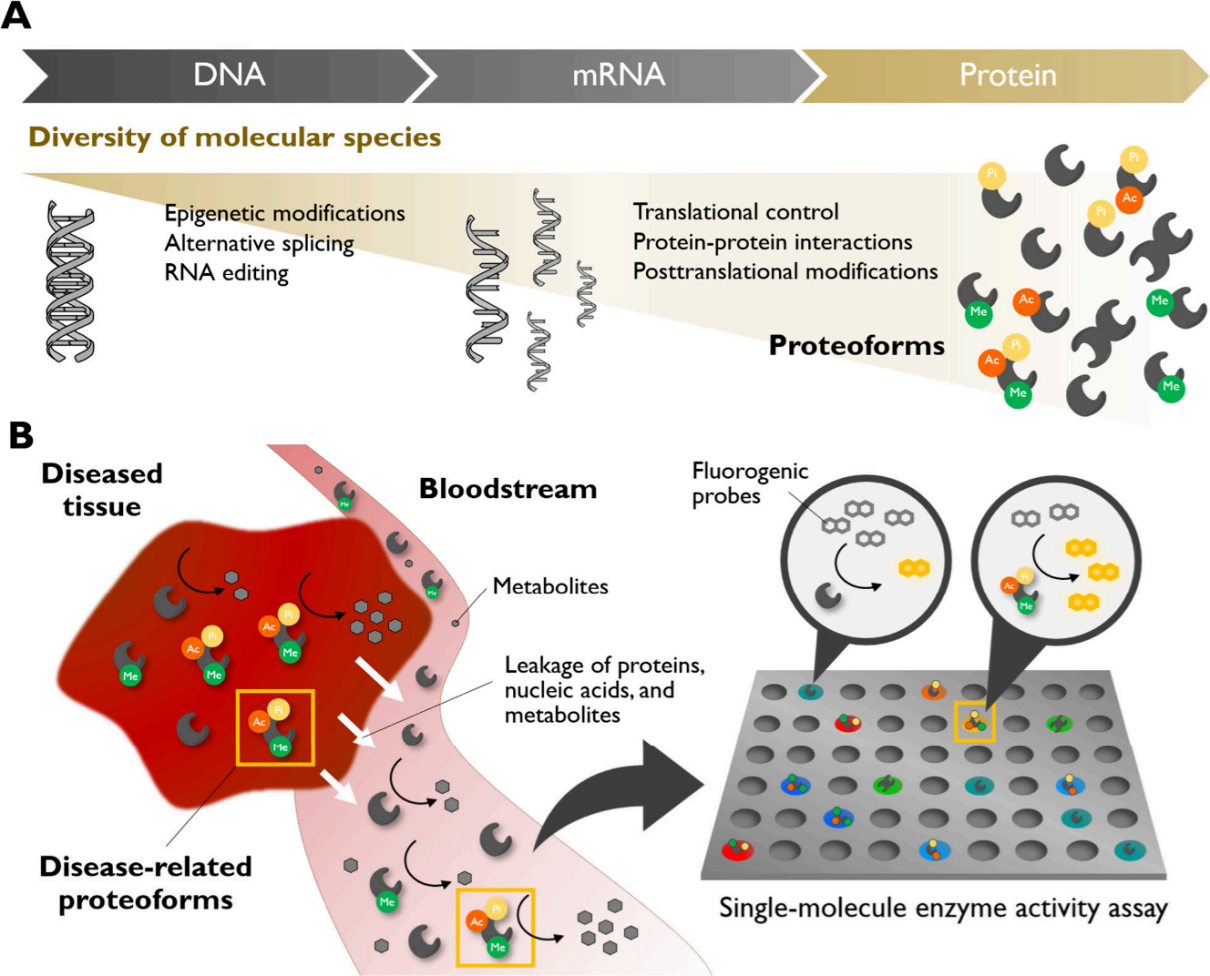
Diversity of the protein world, the concept of proteoforms,^4,5^ and the application of single-molecule enzyme activity analysis for detecting various functional enzyme species in diseases. (A) A diversity of proteoforms are generated from a single gene. (B) The concept and components for detecting disease-related proteoforms in blood samples for highly sensitive and informative activity-based diagnostics.

Differentiating various proteoforms and discovering those that are closely linked to phenotypic changes will lead to the precise understanding of protein functional alterations in disease.^4,5,7,8^ Currently, mass spectrometry is mainly used for this purpose;^7,8^ however, recent studies have shown the potential of single-molecule enzyme activity analysis for understanding enzyme activity landscapes at the proteoform level (Figure 1B).^9–14^ In this Outlook, we provide an overview of the historical context, current developments, and future directions of single-molecule enzyme activity analysis and its applications to disease diagnosis.

## History of Single-Molecule Enzyme Activity Analysis and Assay Mechanisms

Enzymes constitute one of the largest functional protein subgroups, accounting for approximately 20% of the human genome.^15^ Throughout the history of biomarker discovery, various disease-related enzymes, such as alkaline phosphatase (ALP), γ-glutamyl transferase (γ-GTP), lactate dehydrogenase (LDH), and prostate-specific antigen (PSA), have been characterized and are now widely used.^16,17^ Advances in liquid biopsy technologies have sparked increased focus on leveraging functional protein alterations for diagnostics, often referred to as ″activity-based diagnostics″ (Figure 2).^18,19^ However, insufficient protein detection sensitivity has hindered their application at the proteoform level. For enzymes such as ALP and LDH, enzymes from different tissues with distinct PTMs were separately detectable using native electrophoresis,^20,21^ but increasing the resolution to the single-molecule level will further improve the chance of discovering and characterizing unique proteoforms from different tissues and cells (Figure 1B).

**Figure 2:**
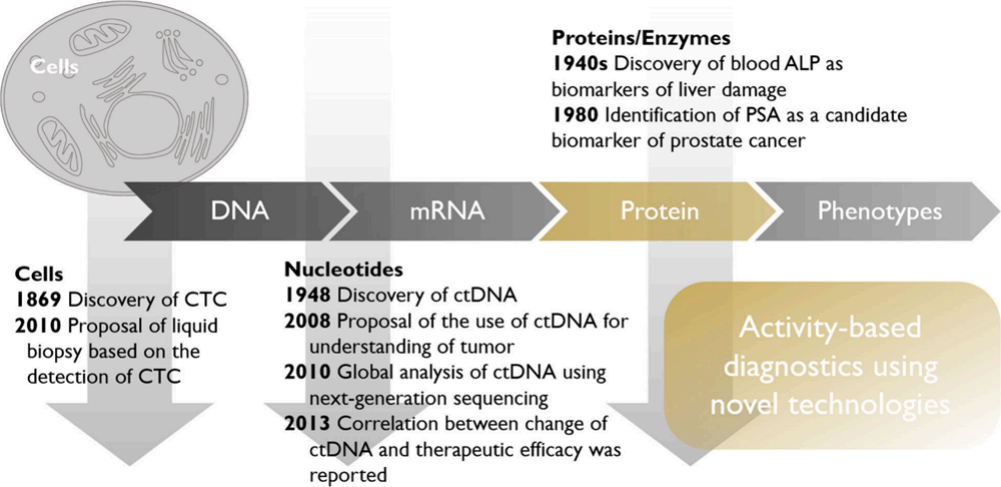
History of liquid biopsy for tumors, focusing on different modalities. The discovery of circulating tumor cells (CTC) dates back to 1869,^77^ and the term “liquid biopsy” was proposed for the detection of CTC in 2010.^78^ The discovery of circulating tumor DNA (ctDNA) dates back to 1948,^79^ and the use of ctDNA and other nucleotide species is considered one of the most promising platforms for liquid biopsy.^80–82^ Protein biomarkers have a longer history;^16,83^ however, only a limited number of technologies have been developed and employed to provide a precise understanding of disease.

Single-molecule enzyme activity analysis has demonstrated its potential in revealing proteoform-level functional alterations of disease-related proteins.

Efforts to analyze enzyme activity at the single-molecule level have advanced in a separate field of research, whose original aim was to understand the heterogeneous behaviors of recombinant proteins in various conditions.^22–29^ In such assays, an enzyme solution is introduced into an array of confined reactors, aiming for stochastic loading such that each chamber contains either zero or one enzyme molecule (Figure 3A). The chambers are coloaded with fluorogenic substrate probes and an enzyme, resulting in the production of a fluorescent signal when the substrate is converted into a fluorescent product by the enzyme. The concept was first proposed by Rotman in 1961,^30^ but the water-in-oil droplet assay, which exhibited heterogeneous sizes, was not suitable for quantitative analysis. In the 2000s, advances in microelectromechanical systems (MEMS)^31^ and microfluidics enabled standardized single-molecule enzyme activity analysis. A landmark study by Rondelez et al. in 2005 ^29^ reported the successful detection and quantification of β-galactosidase and horseradish peroxidase at the single-molecule level. At that time, assays were mainly designed to work with recombinant proteins in the absence of other protein species. Extending these assays to more complex and physiologically relevant biological samples, such as tissue/cell lysates and blood samples, was challenging since the stochastic nature of protein loading hindered the identifying enzyme species in individual chambers.

**Figure 3:**
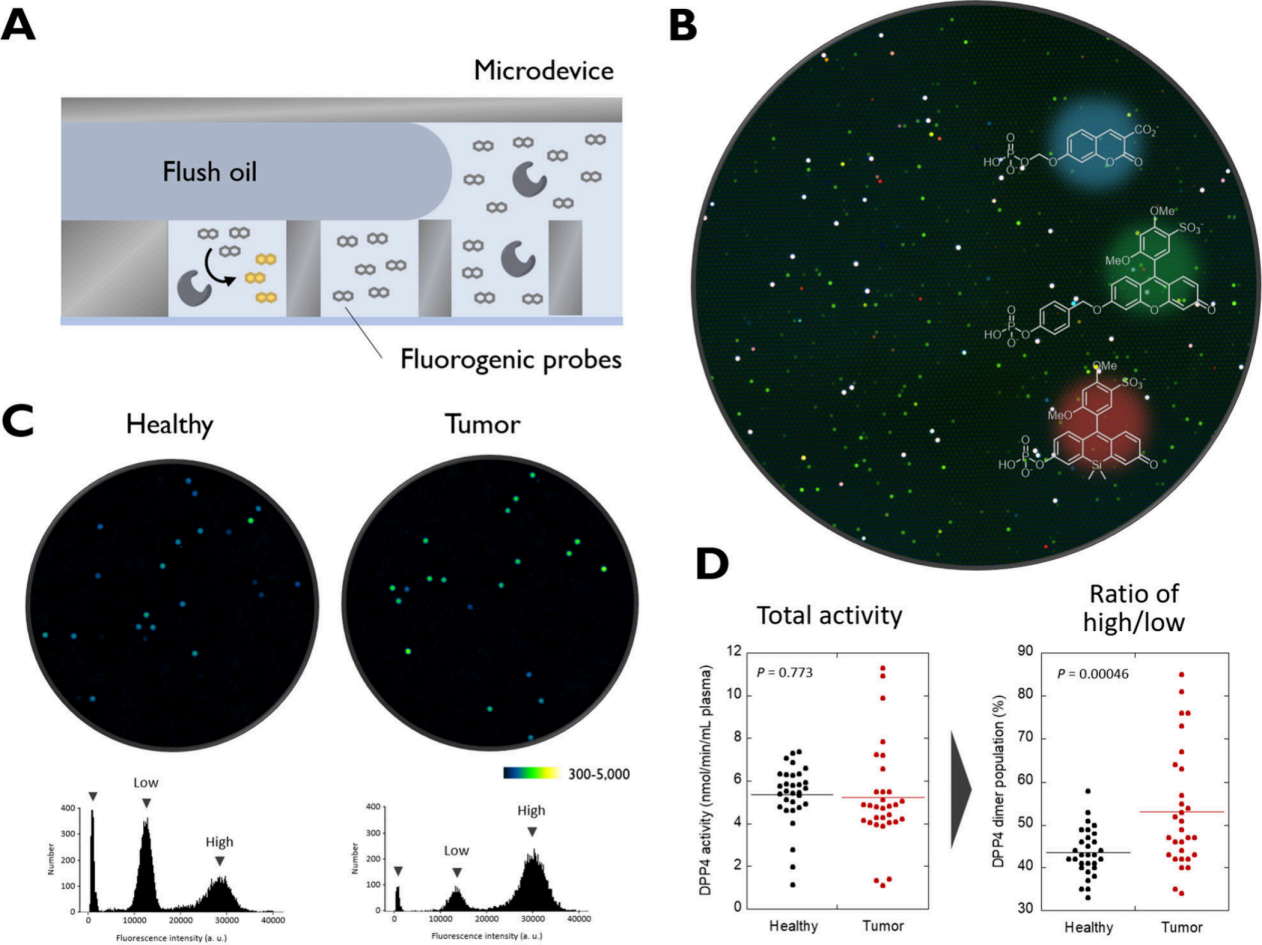
Concept of single-molecule enzyme activity profiling. (A) A schematic representation of single-molecule enzyme activity analysis using a microfabricated chamber device. (B) Single-molecule enzyme activity analysis of various phosphatases in blood samples from healthy human subjects using multicolored and reactivity-wide substrates. The image was acquired under the conditions reported in Sakamoto et al.^9^ (C) Detection of high-and low-activity species of DPP4 in blood samples from healthy subjects (left) and patients with pancreatic cancer (right). In the fluorescence images, spots with greenish colors indicate the high activity species. The histograms show the distribution of single-molecule DPP4 activities. The images were reproduced from Sakamoto et al.^13^ Available under a CC-BY NC license. Copyright 2024. (D) Comparison of total activity of DPP4 (left) and parameters reflecting the population changes of high- and low-activity DPP4 species in single-molecule enzyme activity analysis (right). The images were reproduced from Sakamoto et al.^13^ Available under a CC-BY NC license. Copyright 2024.

The long-standing problem of protein identification in single-molecule enzyme activity assays was recently addressed by a strategy that differentiates proteins based on their unique activity profiles.^9^ In this approach, differences in substrate preferences,^9^ enzyme kinetics,^10^ or inhibition patterns^32^ toward multiple substrates and inhibitors were used to distinguish between enzyme species. In a 2020 study, the proof-of-concept of single-molecule enzyme activity profiling (SEAP) was made by employing multicolored fluorogenic probes to distinctly detect and count various phosphatase species in blood samples (Figure 3B).^9^ The fluorogenic probes were designed to exhibit different reactivities toward various phosphatases, such as subtypes of ALP and protein tyrosine phosphatases, and the activities of individual enzyme molecules were simultaneously recorded in a microfabricated chamber device. The assay successfully detected diverse phosphatase species in blood samples and was able to characterize differences between blood samples of healthy subjects and diabetes patients. An interesting observation made in these studies was the presence of high- and low-activity ALP species in enzymes from the same genetic origin. This phenomenon has also been observed in recombinant enzymes.^33^ While the underlying molecular mechanism remains incompletely understood, progress has been made in recent studies. A 2021 study by Ueno et al. revealed that different activity states arise from the formation of dimers with different activity species, with half a ctivity molecules being heterodimers with inactive monomers lacking proper disulfide bond formation and/or Zn^2+^ incorporation.^34^ Gilboa et al. also explored the mechanisms and focused on changing various environmental factors and discovered that the activity distribution of ALP could be tuned by varying pH and temperature.^35^ These findings indicate that heterogeneous activities reflect differences in substrate-binding site accessibility caused by distinct PTMs or protein substates. Supporting this view, a global mutational analysis reported in 2020 ^36^ showed that heterogeneous ALP activity profiles are an intrinsic property of the protein species, stemming from their diverse substates.

## Application of Single-Molecule Enzyme Activity Analysis to Disease Diagnosis

Following the establishment of single-molecule enzyme activity analysis to complex biological systems, it has served as an ideal platform for activity-based diagnostics due to the high detection sensitivity (Figure S1) and the ability to differentiate proteoforms from different tissues and cells. The 2020 study revealed the elevation of ectonucleotide pyrophosphatase/phosphodiesterase 3 (ENPP3) activity in blood samples of patients with pancreatic cancer.^9^ Due to the low concentration of ENPP3 in blood, conventional fluorescent assays or antibody-based detection were not able to detect the enzyme, whereas single-molecule enzyme activity assays were successful. Following this study, single-molecule enzyme activities were analyzed in various disease-related samples, leading to the discovery of biomarker candidates for liver damage,^10,12,14^ pancreatic tumors,^13^ colorectal tumors,^11^ and brain tumors.^14^

Many discoveries ware made using a hypothesis-driven approach, in which the candidate biomarker is selected from literature knowledge. However, under circumstances where our understanding of single-molecule enzyme activities in blood is limited, it is plausible to employ a screening-type approach, in which a library of assays is prepared to screen patient-derived samples.^2,37,38^ In the 2024 study, activity-based screening using 48 fluorogenic probes targeting different enzymes revealed the proteoform-level alterations in dipeptidyl peptidase 4 (DPP4) activity in blood samples of patients with pancreatic cancer. DPP4 metabolizes the peptide hormone incretin, which stimulates insulin secretion from pancreatic β-cells. While blood DPP4 activity has been studied in various diseases,^39,40^ single-molecule enzyme activity analysis revealed the presence of high- and low-activity species of DPP4 in circulation, whose ratio shifted significantly in the pancreatic tumor group (Figure 3C). Given that DPP4 functions as a dimer,^41^ these findings suggest altered dimer formation in disease. Notably, some patients with early stage pancreatic tumors develop recent-onset diabetes, believed to arise through tumor-induced mechanisms independent of long-standing diabetes.^42^ Since changes in DPP4 activity may influence insulin secretion, further mechanistic studies are warranted.

## Recent Advances in Single-Molecule Enzyme Activity Analysis to Broaden the Targetable Enzymes

While single-molecule enzyme activity analysis has shown its potential in biomarker discovery, the number of enzymes that can be targeted using fluorogenic probes remains limited. As of 2020, single-molecule enzyme activity assays using microfabricated chamber devices were limited to a handful of enzymes due to the scarcity of fluorogenic probes for assay development (Figure 4).^29,30,33,43–46^ Developing suitable fluorogenic probes for single-molecule enzyme activity assays is challenging (Figure S2), but two promising strategies are emerging to develop novel assays: (A) automated synthesis of fluorogenic probes that enables rapid optimization and evaluation, and (B) coupled assays that facilitate activity readouts using natural or physiologically relevant substrates. Below, I summarize the current challenges and explore perspectives for expanding the range of targetable enzymes using these approaches.

**Figure 4:**
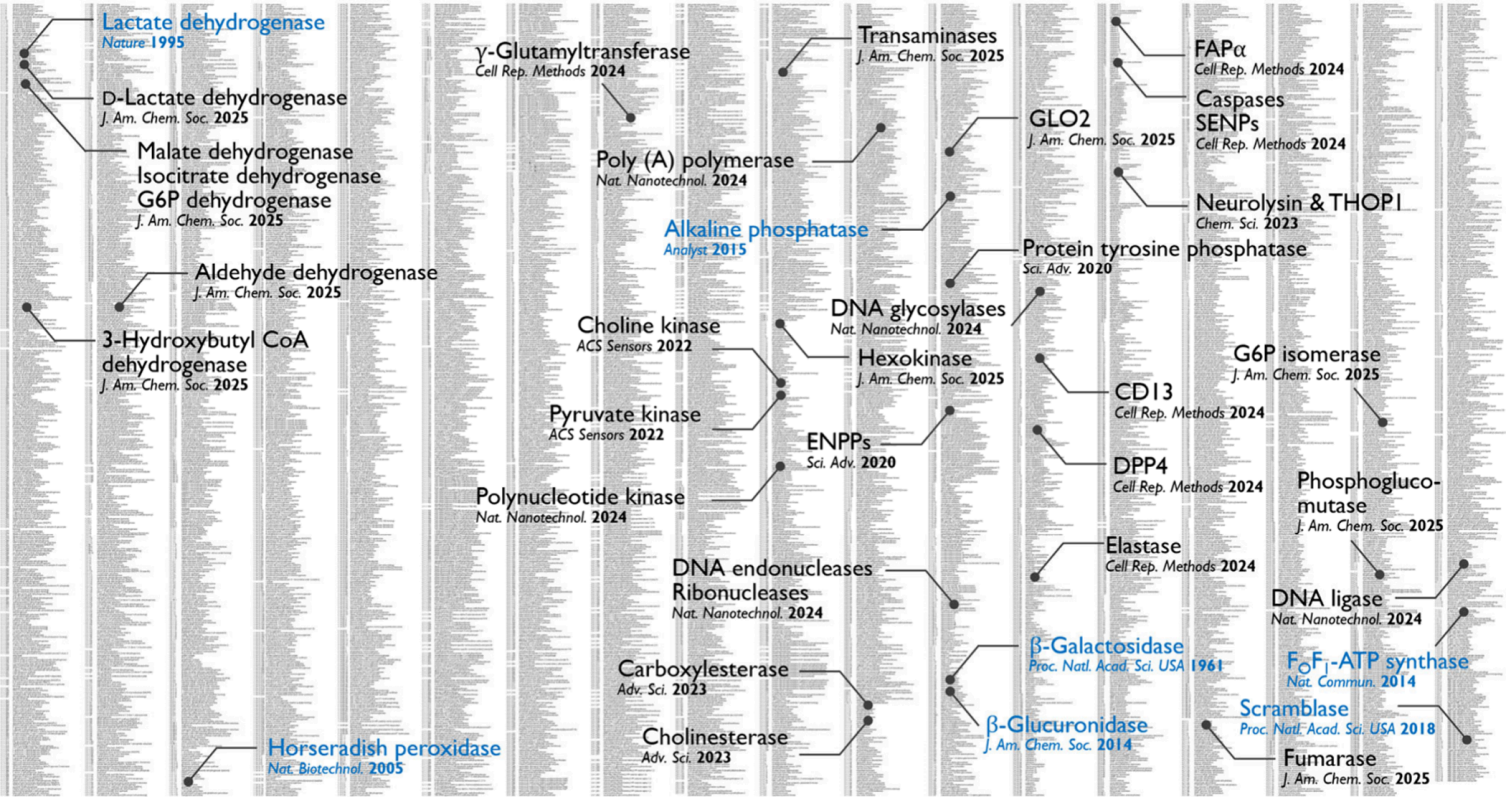
Target enzymes of single-molecule enzyme activity analysis. The enzymes are listed according to their enzyme commission (EC) number and are derived from the BRENDA enzyme database (as of December 1st, 2024). The full list is shown in Figure S3. The single-molecule assays reported before 2020 are marked in blue, with references to the first report.^29,30,33,43–46^ Assays reported after 2020 are marked in black. The assays target various phosphatases,^9^ kinases,^57^ esterases,^12^ peptidases,^11,13^ oxidoreductases,^14^ and DNA processing enzymes.^60^

Recent advancements of the methodologies led to (1) the application of single-molecule enzyme activity assays to study individual proteins in complex biological samples, and (2) expansion of targetable enzymes.

### Automated Probe Synthesis

The limited availability of fluorogenic probes for single-molecule studies highlights the need for specialized strategies to improve probe synthesis. Combinatorial library construction represents a promising approach, and recent developments have extended this concept to automated compound synthesis.^47,48^ Automated synthesis has shown several successes in drug discovery; however, its application in chemical probe development remains limited, partially due to the difficulties of standardizing purification protocols. Chemical probes are not constrained by drug-likeness rules, such as Lipinski’s rule of five,^49^ making its chemical space (i.e., molecular weight and charges) diverse; thus, it is difficult to apply standardized purification protocols. Moreover, turn-on type chemical probes are often chemically unstable and require high purity for accurate activity testing, so stringent and repeated purification is often required.

A feasible method for standardizing the purification of chemical probes is to employ solid-phase synthesis. This method is commonly applied in the preparation of chemical probes with peptide backbones.^38,50^ Here, the reactants are anchored onto a solid support, enabling purification through simple washing steps. However, the scope of solid-phase synthesis is limited to certain reaction types and conditions.^13,38^ An alternative approach is synthesis-based affinity separation (SAS). SAS involves liquid-phase reactions with reactants equipped with an affinity handle, followed by purification through solid-phase affinity separation.^51^ SAS facilitates the synthesis of diverse molecules suitable for combinatorial or automated synthesis by combining the efficiency and versatility of liquid-phase reactions with the ease of purification offered by solid-phase techniques (Figure 5A). This approach has been successfully applied to generate fluorogenic probes for single-molecule activity analysis targeting protease and peptidase activities^13^ (Figure 5B).

**Figure 5:**
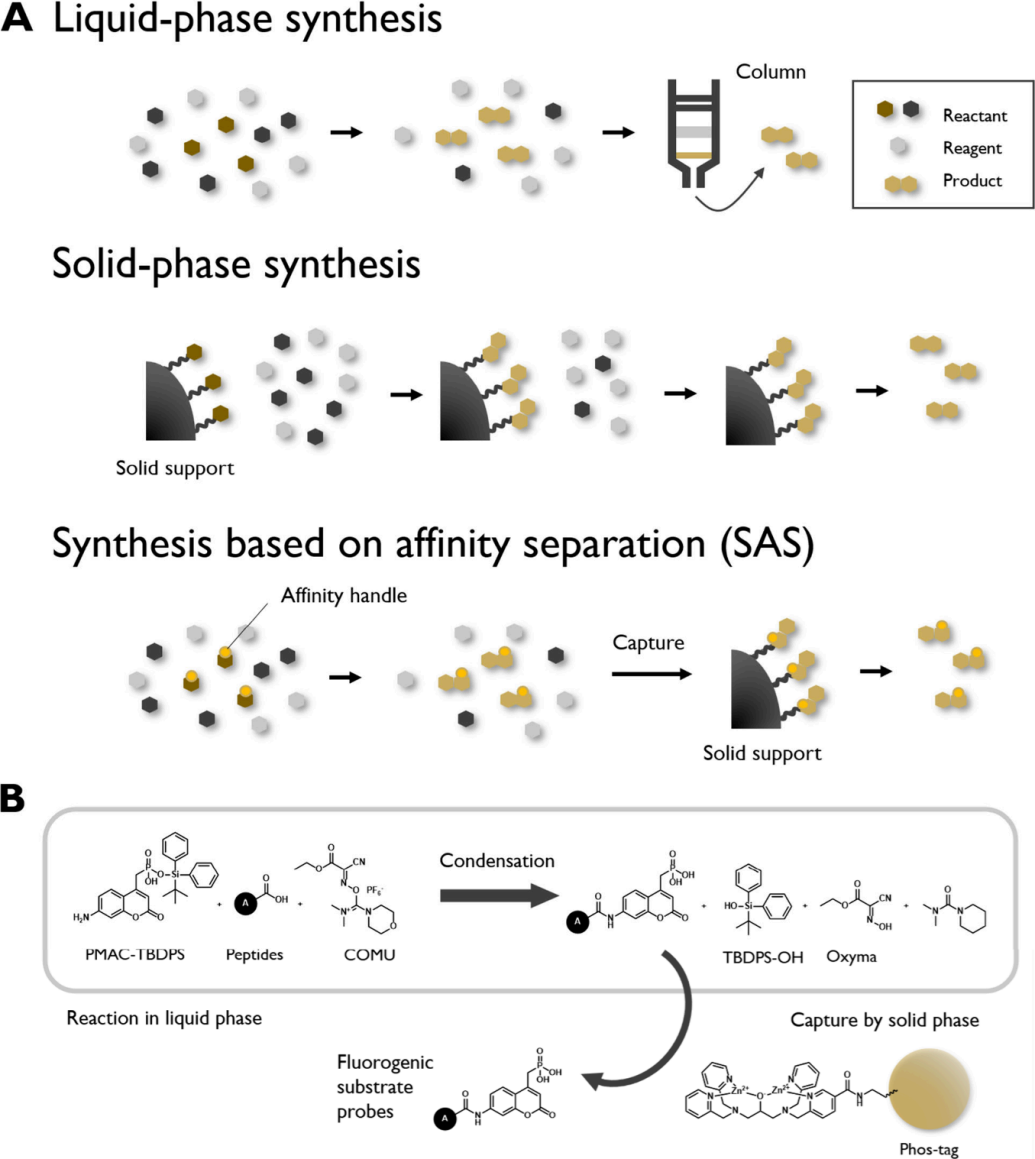
Strategies used for automated synthesis of chemical probes. (a) Schematic representations of various forms of synthesis and purification. In synthesis-based on affinity separation (SAS), the reaction is performed in the liquid phase and purification is performed using a solid support, thus permitting the use of liquid-phase synthesis while the purification follows the principles of solid-phase synthesis. (b) Application of SAS for the preparation of peptidase probes. In this scheme, a phosphonate group is used as an affinity handle. After the amidation reaction, the product can be captured from the reaction mixture using a phos-tag attached to the solid support. The image was reproduced from Sakamoto et al.^13^ Available under a CC-BY NC license. Copyright 2024.

Currently, fluorogenic probes for specific target enzymes are designed based on knowledge of their preferred substrate structures. Automated synthesis is especially powerful when it is accompanied by artificial intelligence (AI)-driven *de novo* molecular design, in which the candidate compounds are designed from the bioactivities of training compounds.^52,53^ In the future, the accumulation of biochemical data will enable *de novo* probe design to create substrate sets capable of detecting diseases or specified proteoforms with greater accuracy.

### Coupled Assays

A coupled assay is a system in which the product of a target enzymatic reaction is detected via a secondary reaction that produces fluorescent signals (Figure 6A). Typically, secondary reactions involve another enzyme as the detection unit, leveraging the high specificity of enzymes to selectively detect the target product.^54^ A key advantage of coupled assays is the ability to use natural or physiologically relevant substrates to detect activity. This makes coupled assays particularly valuable for enzymes that specifically recognize their natural substrates, for which designing fluorogenic substrate analogs is challenging.

**Figure 6:**
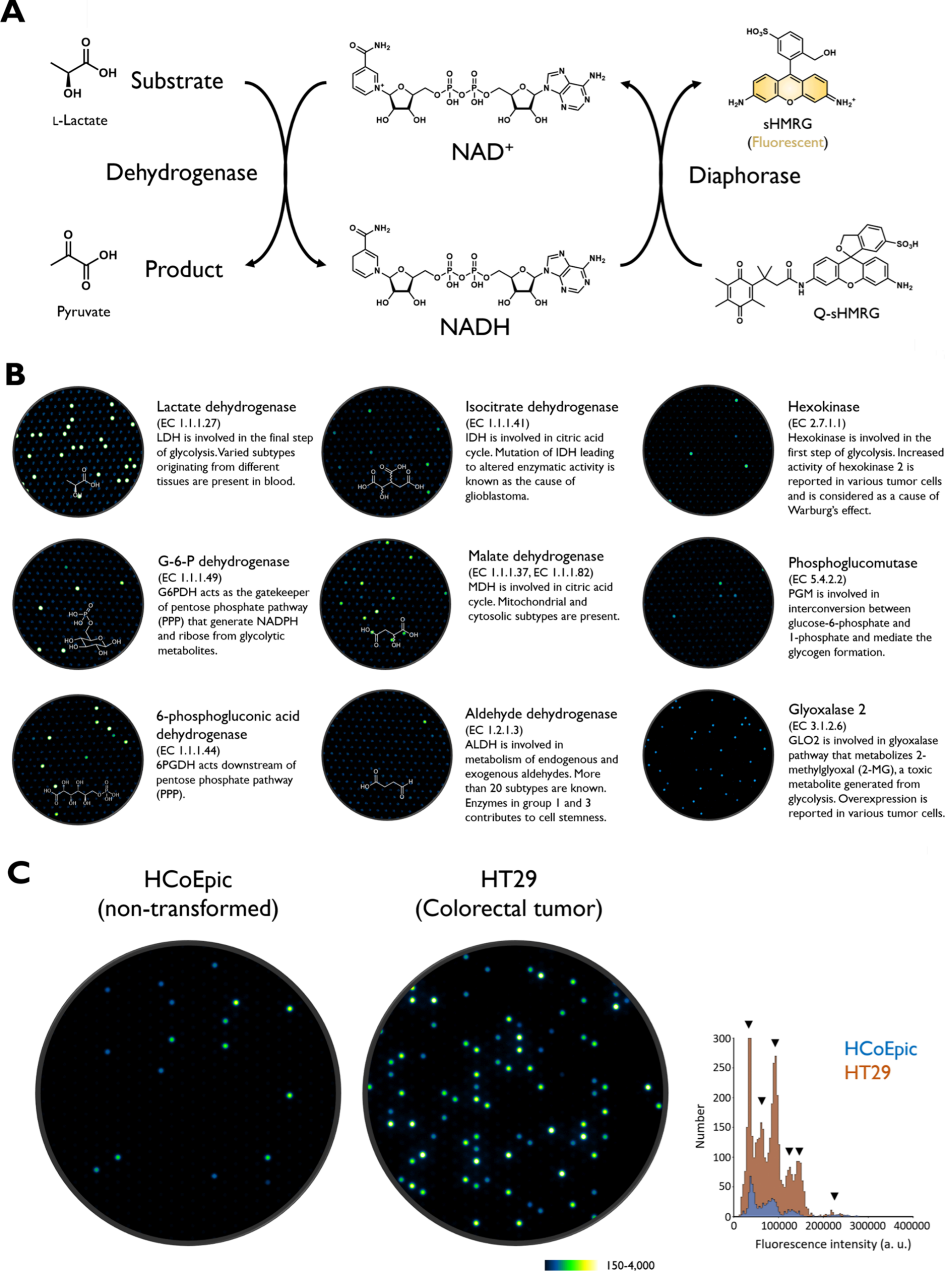
Application of coupled assays for detecting various oxidoreductases at the single-molecule level. The images were reproduced from Minoda et al.^14^ Copyright 2025, American Chemical Society. (A) Schematic of the coupled assay for single-molecule oxidoreductase activity analysis. (B) Representative enzymes detectable at single-molecule level using the coupled assay scheme. The fluorescence images show the results of activity detection in blood samples from healthy human subjects. (C) Detection of single-molecule activity distributions of G6PDH in lysates of HCoEpic (nontransformed colorectal epithelial) cells and HT29 (colorectal tumor) cells.

Oxidoreductases are enzymes that catalyze the reduction or oxidization of substrates. Since many oxidoreductases use NAD­(P)^+^ as a cofactor and generate NAD­(P)H upon oxidizing their substrates,^14^ coupled assays to detect NAD­(P)H to measure various oxidoreductase activities have been developed.^54–56^ Recently, this assay scheme was adapted to detect oxidoreductase activities at the single-molecule level (Figure 6B),^14,57^ and many enzymes involved in central metabolic pathway^58^ became monitorable in this platform. For example, glucose-6-phosphate dehydrogenase (G6PDH) is a gatekeeper of the pentose-phosphate pathway (PPP) to supply intermediates of nucleic acid synthesis.^58^ G6PDH activity is often altered in rapidly proliferating tumor cells through changes in expression levels or modulation by various PTMs.^59^ Single-molecule enzyme activity analysis of lysates from various colorectal tumor and nontumor cells has revealed diverse activity species present within each cell type, and some activity species were uniquely observed in tumor cells (Figure 6C).^14^ While the underlying molecular mechanism is under investigation, the result indicated the potential of the single-molecule enzyme activity assay to detect cell type-specific proteoform landscapes of targeted cells for understanding and detecting disease (Figure 1B). Single-molecule G6PDH activity was also measured in the cerebrospinal fluid (CSF) of patients with brain tumors, demonstrating its potential as a biomarker to estimate tumor mass.^14^

Another example of a coupled assay is the use of thioester as a structural analogue of ester, and single-molecule esterase activities were measured by detecting thiol generated from thioester hydrolysis.^12^ Single-molecule activities of various esterases including cholinesterases, enzyme with strict substrate specificity, were successfully studied, and the changes in single-molecule butyrylcholinesterase activity were detected in blood samples of mice with acute liver damage.^12^ Recently, Gines et al. reported a generalizable single-molecule enzyme activity assay to study various DNA/RNA-metabolizing enzymes.^60^ The system employed DNA-enzyme circuits to detect the formation of targeted DNA in water-in-oil droplets. The advantage of the system is that the DNA formation signal is amplified in the droplet, enabling the detection of slow-reacting enzymes such as Cas9. The assay platform was able to detect various DNA/RNA-metabolizing enzymes such as DNA/RNA nucleases, DNA glycosylases, poly­(A) polymerase, DNA kinase and DNA ligase.^60^ As with other coupled assays, once the detection system is established, it can be readily adapted to new targets, and further applications expanding the repertoire of targetable enzymes are highly anticipated.

## Future Perspectives – Expansion of the Systems for General Applications

In genomic and transcriptomic analysis, the advent of next-generation sequencing (NGS) revolutionized the field by dramatically increasing throughput and data depth compared to Sanger sequencing and microarray technologies.^61^ NGS leveraged the power of single-molecule analysis for highly parallel profiling of target molecules.^62,63^ Analogously, the development of single-molecule protein analysis platforms holds transformative potential for deepening our understanding of protein function at the proteoform level.

The unique biomarker candidates for various diseases, such as pancreatic tumors, colorectal tumors, brain tumors, and liver damage have been discovered using newly developed single-molecule enzyme activity assays.

In this review, we have primarily focused on advances in single-molecule “enzyme” activity analysis. However, protein function extends beyond enzymatic catalysis, encompassing transport, binding, and structural roles. Single-molecule assays targeting these functions, such as patch-clamp methods for ion transport,^64^ evanescent wave fluorescence microscopy^65^ and zero-mode waveguides^66^ for binding, also offer promising avenues to explore proteoform-level functional diversity within complex biological systems.

Another emerging technology for next-generation protein analysis is single-molecule sequencing of proteins. Various strategies are under development, including Edman degradation-based methods,^67^ nanopore-based sequencing,^68–71^ and approaches using *N*-terminal amino acid-binding proteins.^72^ However, “sequence” and “function” provide fundamentally different layers of information. Thus, functional and sequence-based analyses will likely coevolve as complementary technologies, much like the relationship between biochemical assays (functional analysis) and mass spectrometry (sequence analysis). Bridging these, *in situ* structural analysis at the single-molecule level is also becoming possible. In 2023, Chen et al. demonstrated the identification of individual protein molecules in mammalian sperm by fitting *in situ* cryo-electron tomography (cryo-ET) density maps to AlphaFold2-predicted structures.^73^ Although this approach does not yet resolve molecular individuality, it highlights the potential to visualize entire protein molecules with near-atomic resolution.

Among these emerging approaches, single-molecule enzyme activity analysis holds strong promise for contributing to next-generation protein analysis. Nevertheless, several challenges must be addressed to fully realize its potential. A major limitation is the limited scope of enzymes that can be targeted. Although recent advances have expanded the available assay repertoire, many biologically important enzymes remain untargetable due to the lack of appropriate detection schemes or inherently low catalytic turnover. This gap is expected to close with continued innovations in probe and biosensor development.^17,74^ Data interpretation is another important challenge. Studies of well-characterized enzymes, such as β-galactosidase and alkaline phosphatase, have begun to reveal connections between heterogeneous single-molecule activities and the underlying molecular mechanisms.^22–25,27–29,34–36^ However, current understanding is limited and the generalizability of these findings across diverse enzymes remains unclear. In the future, nonbiased, AI-driven data analysis methods could be effective in uncovering hidden correlations between molecular states and phenotypes, as seen in flow cytometry^75^ or fluorescence imaging.^76^ This AI-based analyses will require large experimental data sets from single-molecule enzyme activity assays that capture vast amounts of information reflecting proteoform-level changes, including variations in isoforms, mutations, PTMs, and conformational substates.

Further expansion of the targetable enzymes and co-development of data analysis platforms will advance the assay to serve as an important pillar of next-generation protein functional analysis.

By addressing these challenges, single-molecule enzyme activity analysis could emerge as a cornerstone technology for next-generation protein functional analysis, opening new frontiers in both basic biology and translational research in healthcare.

## Supplementary Material



## Notes

Equations for evaluating the performance of single-molecule enzyme activity analysis using the microfabricated chamber device, requirement of characteristics of fluorogenic probes for single-molecule enzyme activity assays, and a list of enzymes in the BRENDA enzyme database (PDF)
