# Supplementary material for: Single-Molecule Enzyme Activity Analysis for Illuminating Pathological Proteoforms
Source: ACS Cent Sci. 2025 Jun 17;11(7):1041–51. doi: 10.1021/acscentsci.5c00100 (PMC12291115; doi:10.1021/acscentsci.5c00100)
Supplement: Supplementary file 1 [file oc5c00100_si_001.pdf]

Supporting Information

Single-molecule enzyme activity analysis for illuminating pathological proteoforms

Toru Komatsu and Tadahaya Mizuno

Graduate School of Pharmaceutical Sciences, The University of Tokyo,

7-3-1 Hongo, Bunkyo-ku, Tokyo 113-0033, Japan

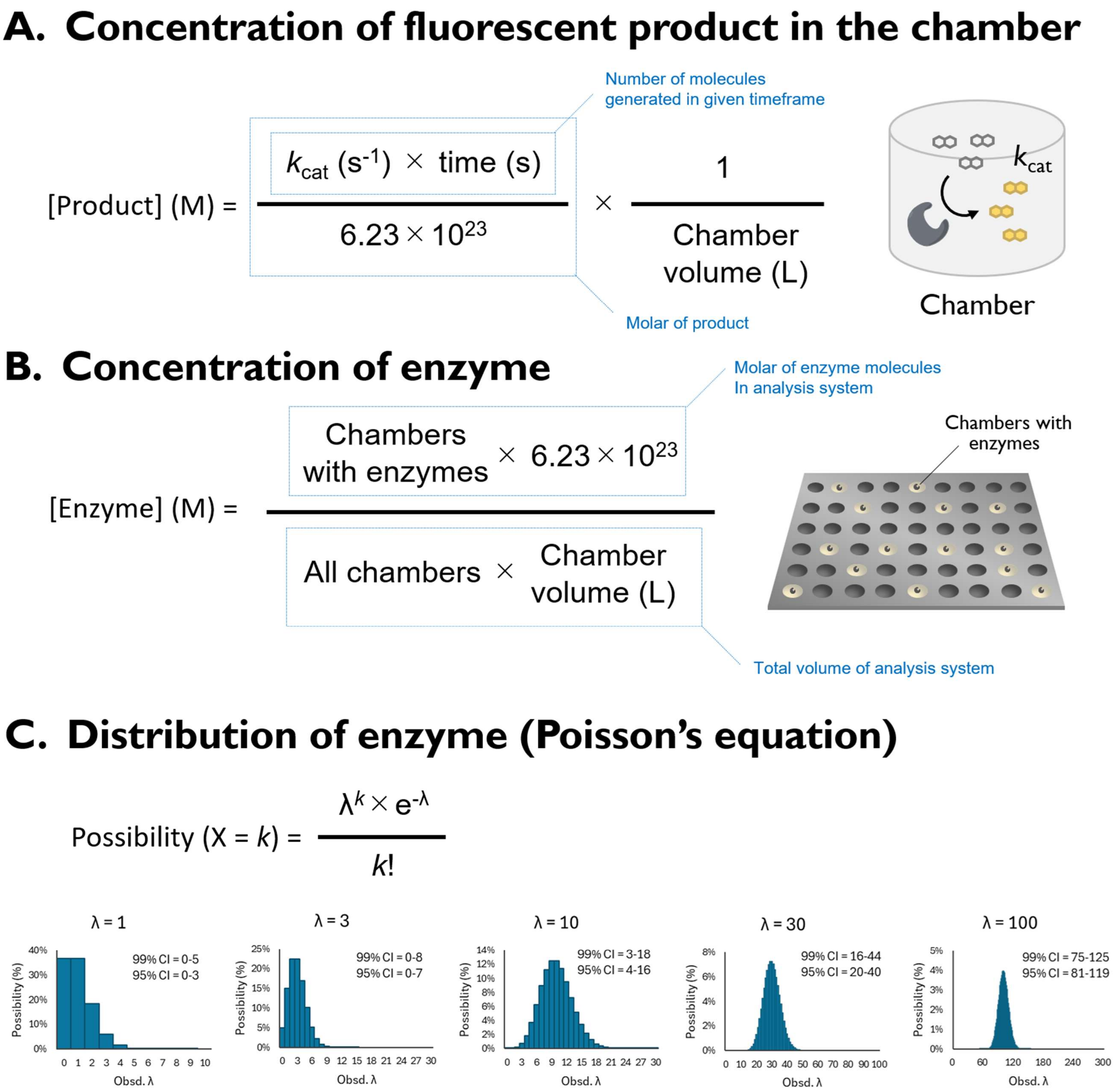

Figure S1. Equations used to evaluate the performance of single-molecule enzyme activity analysis using the microfabricated chamber device. (A) Equation used to estimate the concentration of fluorescent product produced by turnover-based metabolism of substrates. (B) Equations used to estimate the concentration of the enzyme in the sample. (C) Equation used to estimate the distribution of the expected number of spots in single-molecule analysis (observed spot number; Obsd. λ) from the true value (λ)[1]. CI indicates the confidence interval.

[Comment]

The single-molecule enzyme activity detection relies on the enzyme turnover generating the fluorescent product in the small chamber, so if the turnover number (k_{cat}) is small, it becomes difficult to acquire sufficient fluorescent signals. The equation in **Figure S1A** shows that a higher turnover number (k_{cat}) of enzymes or longer incubation time linearly affects the signal intensity (reflection of the concentration of the product). In addition, the small volume of the chamber can contribute to increased fluorescence signal. For example, in detecting the enzyme with a turnover rate (k_{cat}) of 1 s^{-1}, it can generate 3,600 molecules or 6 zmol of fluorescent products over 1 h. This amount of product would be too low to be detected in the standard analysis methodologies, but within a confined volume of 50 fL, the local concentration of the fluorescent product can reach 120 nM, which can give sufficient fluorescent signal in a conventional fluorescence microscope (**Figure S2**)[2]. The calculation tells us that it is challenging to detect the enzymes with lower k_{cat} values, but the database search indicated that over 50% of the enzymes exhibit k_{cat} > 1 s^{-1} for their physiological substrates[3]; therefore, we can expect that the assay can be widely applicable to various enzymes at if the proper readout system can be constructed.

The equation in **Figure S1B** is useful to discuss the detection sensitivity of single-molecule enzyme activity assay. While the assay can detect the target if one molecule of enzyme is present in the system, the practical detection limit is governed by the possibility of capturing the desired target in the reaction chamber. Current assay protocols typically involve the simultaneous analyses of 10^5-10^6 chambers. In analyzing 10^6 chambers with 50 fL volume, when we detect 100 chambers that contain target enzymes, the estimated concentration is 3.3 fM (0.17 pg/mL, if the molecular weight is 50,000). Increasing the number of chambers is a straightforward way to lower the detectable concentration. The increased volume also enhances the chance of capturing the target molecule, but this is the tradeoff with the weakening of signal intensity acquired from the single-molecule enzyme (from the equation (A)).

The equation in **Figure S1C** is used to discuss the stochastic nature of loading the target protein in the chamber. It tells us that we need to be aware of the randomness if we treat a small number of molecules (i.e. less than 100 molecules detected in one assay). For example, when the true spot number (chambers with enzymes) corresponds to 10, the observed spot number will be between 4-16 with a 95% possibility, giving high variations of measurement results. In accordance with **Figure S1B**, if one wants to detect enzymes with lower concentrations, it is recommended to increase the number of chambers analyzed in each assay to ensure that more than 100 enzyme molecules are detected per assay.

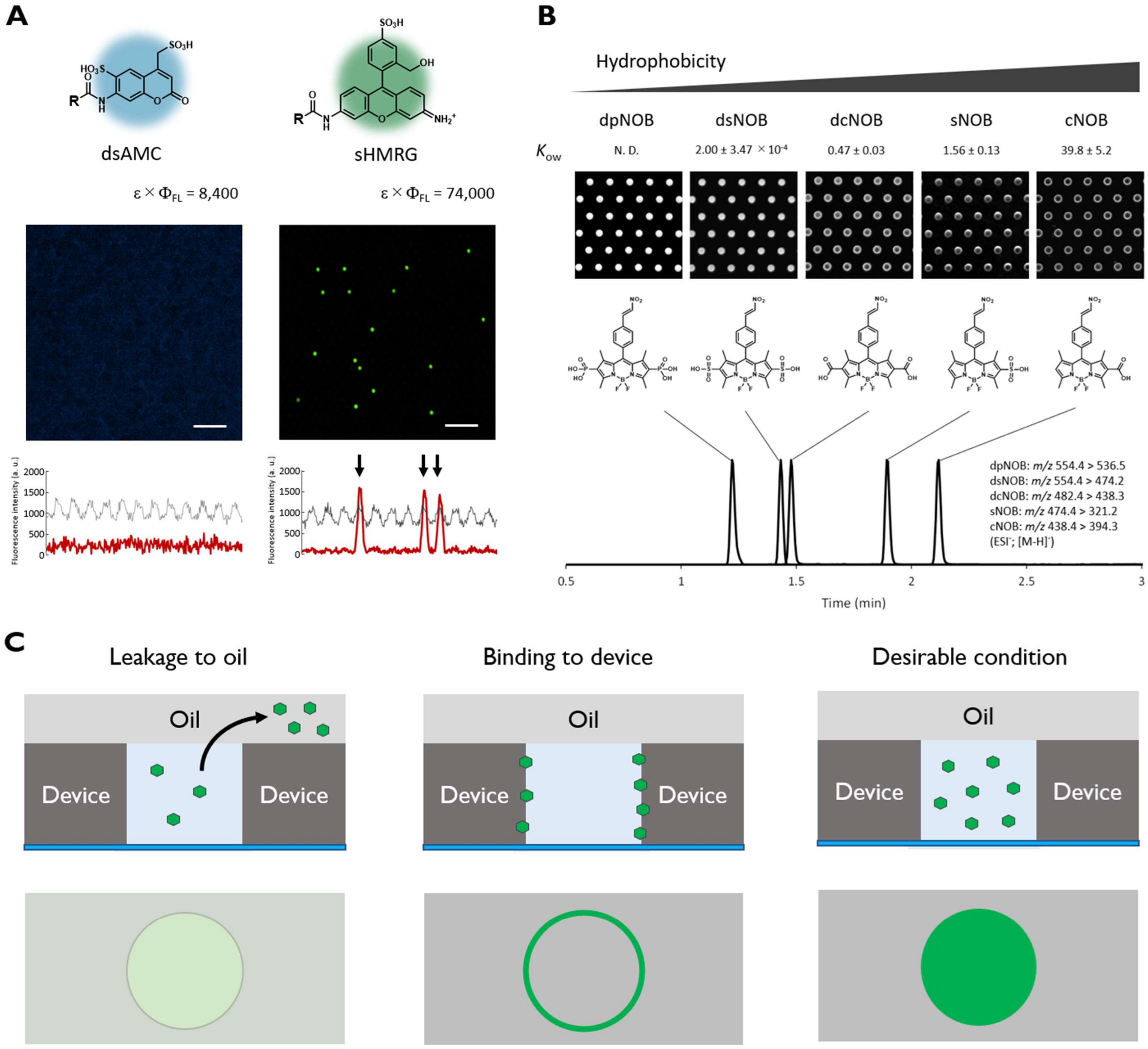

Figure S2. Data supporting the importance of brightness and hydrophilicity for performing single-molecule enzyme activity assays. (A) The results of detecting lactate dehydrogenase (LDH) activity in microdevice using the coumarin-based (left) or rhodamine-based (right) fluorogenic probes. R = 3-Methyl-3-(2,4,5-trimethyl-3,6-dioxocyclohexa-1,4-dien-1-yl)butanoic acid. $\varepsilon \times \Phi_{FL}$ was calculated from the values of 7-amino-4-methylcoumarin and rhodamine green[4,5], respectively. The images were reproduced from reference[2]. Copyright 2025, American Chemical Society. (B) Applicability of BODIPY-based fluorophores with various hydrophilic substituents for the microdevice-based assays. K_{OW} indicates the *n*-octanol/water partition coefficient. The images were reproduced from reference[6]. Available under a CC-BY NC license. Copyright 2023. (C) Possible negative effects (leakage to oil or binding to device) occurring in single-molecule enzyme assays as a result of insufficient hydrophilicity of the probes. The images were reproduced from reference[6]. Available under a CC-BY NC license. Copyright 2023.

[Comment]

The development of fluorogenic probes for the microdevice-based analyses is challenging as they must meet at least three criteria: (1) rapid and selective reactivity with the target enzyme, (2) a high signal-to-noise ratio and bright fluorescence, and (3) sufficient hydrophilicity to ensure compatibility with microdevice-based assays[2,6]. The criteria (1) and (2) are particularly important for detecting weaker enzyme activity, since, even when enough product is generated in the chamber, the detectability of the fluorescent signal relies on the brightness of the fluorophore and signal-to-noise ratio. For example, in the experiments in **Figure S2A**, use of bright fluorophore with excitation at visible light region (rhodamine-based, right) was essential for detecting single-molecule lactate dehydrogenase (LDH) activity in the microdevice[2]. The probe with UV-excitable coumarin fluorophore (left) suffered from poor UV light penetration and low brightness (represented by $\varepsilon \times \Phi_{FL}$) of the fluorophore.

As for the criteria (3), the probe with insufficient hydrophilicity was unsuitable for the microdevice-based assay since the hydrophobic fluorophore can leak out of system or binding to the microdevice (**Figure S2B**, **S2C**)[6,7]. Introducing proper hydrophilic functional groups, such as phosphonate and sulfonate, is desirable to increase the hydrophilicity of the fluorophore[6].

Rerefences

(1) Rissin, D. M.; Walt, D. R. Digital Concentration Readout of Single Enzyme Molecules Using Femtoliter Arrays and Poisson Statistics. *Nano Lett.* **2006**, *6* (3), 520–523. https://doi.org/10.1021/nl060227d.

(2) Minoda, M.; Hatakeyama, J.; Nagano, N.; Mizuno, T.; Iwasaka, T.; Shiga, S.; Takahashi, K.; Hiraide, H.; Sakamoto, S.; Kagami, Y.; Kashiro, A.; Honda, K.; Sugiura, Y.; Mishima, K.; Mishima, M. K.; Kusuhara, H.; Urano, Y.; Komatsu, T. Single-Molecule Oxidoreductase Activity Analysis for Activity-Based Diagnosis Based on Proteoform Alterations. *J. Am. Chem. Soc.* **2025**, *147* (6), 4743–4751. https://doi.org/10.1021/jacs.4c07624.

(3) Bar-Even, A.; Noor, E.; Savir, Y.; Liebermeister, W.; Davidi, D.; Tawfik, D. S.; Milo, R. The Moderately Efficient Enzyme: Evolutionary and Physicochemical Trends Shaping Enzyme Parameters. *Biochemistry* **2011**, *50* (21), 4402–4410. https://doi.org/10.1021/bi2002289.

(4) Kubin, R. F.; Fletcher, A. N. Fluorescence Quantum Yields Of Some Rhodamine Dyes. *J. Luminescence* **1982**, *27*, 455–462.

(5) Ranganathan, R.; Lenti, G.; Tassone, N. M.; Scannell, B. J.; Southern, C. A.; Karver, C. E. Design and Application of a Fluorogenic Assay for Monitoring Inflammatory Caspase Activity. *Anal. Biochem.* **2018**, *543*, 1–7. https://doi.org/10.1016/j.ab.2017.11.023.

(6) Ukegawa, T.; Komatsu, T.; Minoda, M.; Matsumoto, T.; Iwasaka, T.; Mizuno, T.; Tachibana, R.; Sakamoto, S.; Hanaoka, K.; Kusuhara, H.; Honda, K.; Watanabe, R.; Urano, Y. Thioester-Based Coupled Fluorogenic Assays in Microdevice for the Detection of Single-Molecule Enzyme Activities of Esterases with Specified Substrate Recognition. *Adv. Sci.* **2023**, *11* (10), 2306559. https://doi.org/10.1002/advs.202306559.

(7) Sakamoto, S.; Komatsu, T.; Watanabe, R.; Zhang, Y.; Inoue, T.; Kawaguchi, M.; Nakagawa, H.; Ueno, T.; Okusaka, T.; Honda, K.; Noji, H.; Urano, Y. Multiplexed Single-Molecule Enzyme Activity Analysis for Counting Disease-Related Proteins in Biological Samples. *Sci. Adv.* **2020**, *6* (11), eaay0888. https://doi.org/10.1126/sciadv.aay0888.

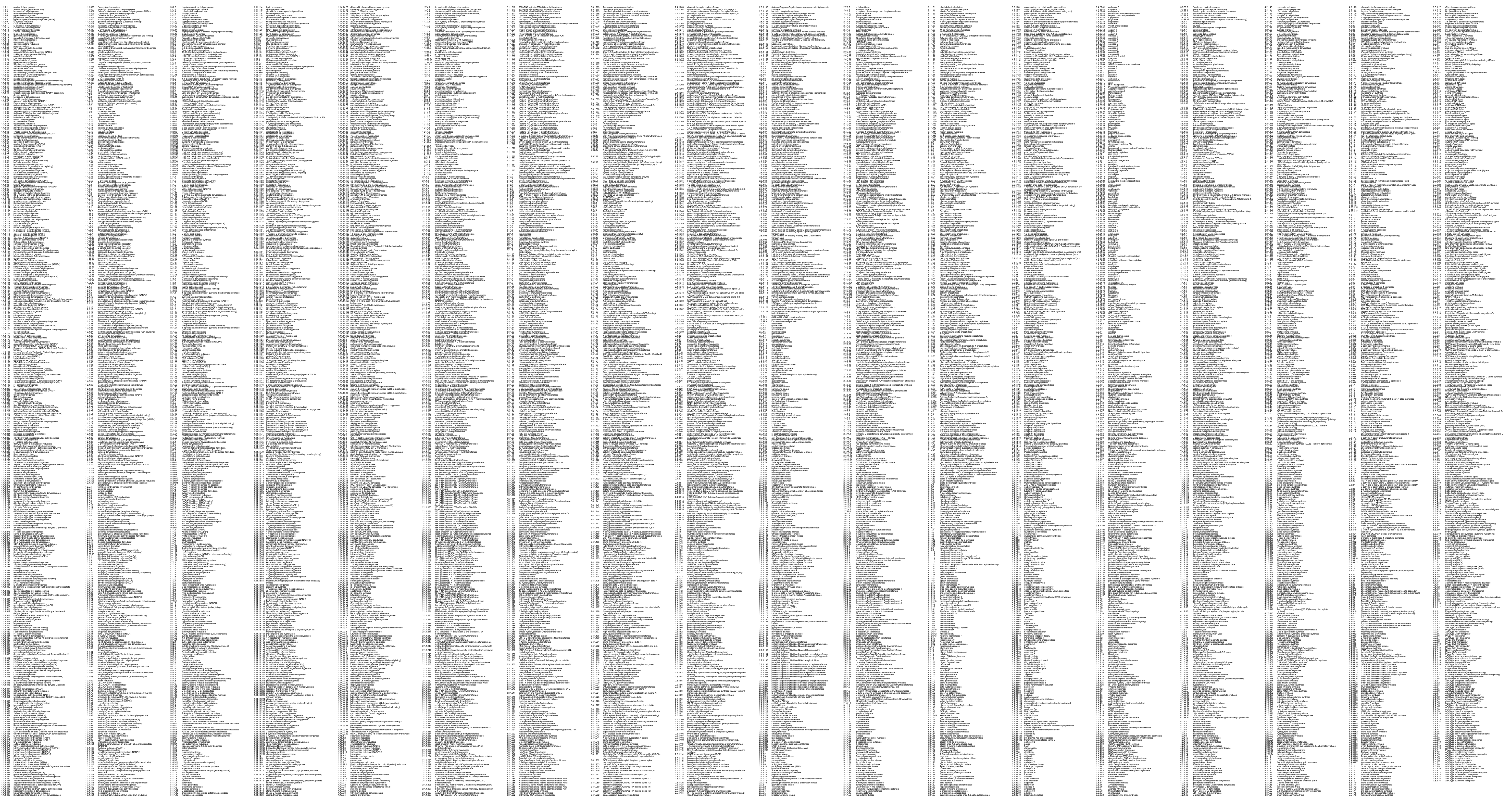

Figure S3. List of enzymes in the BRENDA enzyme database (as of December 1st, 2024).
